# Seed priming with proline improved photosystem II efficiency and growth of wheat (*Triticum aestivum* L.)

**DOI:** 10.1186/s12870-021-03273-2

**Published:** 2021-10-30

**Authors:** Sarah Ambreen, Habib-ur-Rehman Athar, Ameer Khan, Zafar Ullah Zafar, Ahsan Ayyaz, Hazem M. Kalaji

**Affiliations:** 1 Department of Botany, University of Sargodha, Sargodha, 40100 Pakistan; 2 Department of Botany, Bahauddin Zakariya University, Multan, 60800 Pakistan; 3 Department of Botany, Division of Science and Technology, University of Education Lahore, Lahore, Punjab 54770 Pakistan; 4 Institute of Crop Sciences, Zhejiang University, Hangzhou, China; 5 Department of Plant Physiology, Institute of Biology, University of Life Sciences SGGW, 02-776 Warsaw, Poland; 6 Institute of Technology and Life Sciences - National Research Institute, Falenty, Al. Hrabska 3, 05-090 Raszyn, Poland

**Keywords:** OJIP, Performance index, Quantum yield of PSII, Oxygen evolving complex

## Abstract

**Background:**

Proline can promote growth of plants by increasing photosynthetic activity under both non-stress and abiotic stress conditions. However, its role in non-stressed conditions is least studied. An experiment was conducted to assess as to whether increase in growth of wheat due to seed priming with proline under non-stress condition was associated with proline-induced changes in photosystem II (PSII) activity. Seeds of four wheat varieties (S-24, Sehar-06, Galaxy-13, and Pasban-90) were primed with different concentrations of proline (0, 5, 15 and 25 mM) for 12 h and allowed to grow under normal conditions. Biomass accumulation and photosynthetic performance, being two most sensitive features/indicators of plant growth, were selected to monitor proline modulated changes.

**Results:**

Seed priming with proline increased the fresh and dry weights of shoots and roots, and plant height of all four wheat varieties. Maximum increase in growth attributes was observed in all four wheat varieties at 15 mM proline. Maximum growth improvement due to proline was found in var. Galaxy-13, whereas the reverse was true for S-24. Moreover, proline treatment changed the Fo, Fm, Fv/Fo, PI_ABS_, PI_Tot_ in wheat varieties indicating changes in PSII activity. Proline induced changes in energy fluxes for absorption, trapping, electron transport and heat dissipation per reaction center indicated that var. Galaxy-13 had better ability to process absorbed light energy through photosynthetic machinery. Moreover, lesser PSII efficiency in var. S-24 was due to lower energy flux for electron transport and greater energy flux for heat dissipation. This was further supported by the fact that var. S-24 had disturbance at acceptor side of PSI as reflected by reduction in ΔV_IP_, probability and energy flux for electron transport at the PSI end electron acceptors.

**Conclusion:**

Seed priming with proline improved the growth of wheat varieties, which depends on type of variety and concentration of proline applied. Seed priming with proline significantly changed the PSII activity in wheat varieties, however, its translation in growth improvement depends on potential of processing of absorbed light energy by electron acceptors of electron transport chain, particularly those present at PSI end.

## Background

In recent scenario of food security and climate change, there remains a constant urge to imply crop improvement strategies. One of these strategies is exogenous use of plant growth regulators that are capable to promote plant growth e.g. hormones or compatible solutes (glycine betaine and proline) as foliar spray or seed priming [1]. Seed priming helps in early seedling growth by expediting the pre-occurrence of metabolic events necessary for seed germination and hence, reduces the time-gap between seed sowing and seedling emergence, improves tillering and grain yield [2, 3]. It cause the over-expression of genes involved in biochemical pathways [4]. Priming mainly targets lag phase of seed germination and accelerates gene expression, rate of DNA repair, activation of enzymes and metabolite accumulation [4]. Seed priming initiates cross tolerance mechanisms which affects plant growth even at later stages of plant growth [3] as observed in wheat, canola, *Capsicum annum*. Some reports suggested that seed priming enhanced the seedling growth by increasing proline and activity of superoxide dismutase (SOD) [5]. Seed priming with proline also improved the growth by improving uptake of mineral nutrients and photosynthesis in canola [2]. Similarly, seed priming alleviated the adverse effects of cadmium stress by enhancing photosystem II activity and antioxidant potential of tomato plants [6]. Long ago, it has been reported that 26% proline pool out of total amino acids was found in seeds of *Arabidopsis*, whereas only 1–3% proline pool was found in vegetative tissues indicating that proline has major role in seed metabolism [7]. Moreover, additional supply of proline via seed priming can be more beneficial in resource management during seed germination, seedling growth and even at later growth stages [8]. For example, exogenous application of L-proline increased the availability of nitrogen for cellular metabolism that helps in plant growth [9]. Proline is involved in regulating photosynthetic machinery [10], and can avert stomatal limitation for fixation of CO_2_. In addition, proline metabolism influence the oxygen pentose phosphate pathway (OPPP), which is crucial for seed germination and seedling growth [11, 12].

Previous published reports suggested that efficacy of exogenously applied proline in enhancing plant growth is highly concentration dependent [13]. Generally, low concentrations of L-proline enhance plant growth while higher concentrations are found to be toxic [14]. Exogenous proline if supplied in higher concentration inhibits P5CS enzyme and generate reactive oxygen species resulting in inhibition of growth in Arabidopsis plants [15, 16]. Some studies suggested that endogenous 20 mM proline can completely deactivate singlet oxygen [17].

These reports suggested that exogenous application of proline can enhance the endogenous level of proline which can modulate variety of physiological and biochemical processes thereby resulting in improved growth. However, it is not yet known which physiological process have major contribution in improving growth. Moreover, optimum proline concentration for seed priming in wheat is also not known. In view of these reports, it is hypothesized that exogenous supply of proline as seed priming can boost seedling growth by modulating photosynthetic capacity. Thus, this study was aimed to optimize proline dose for seed priming in different wheat varieties. Moreover, as to whether proline induced changes in PSII activity (measured as fast chlorophyll *a* fluorescence, OJIP followed by JIP-test) translated in improved growth was also assessed.

## Results

**Fig. 1:**
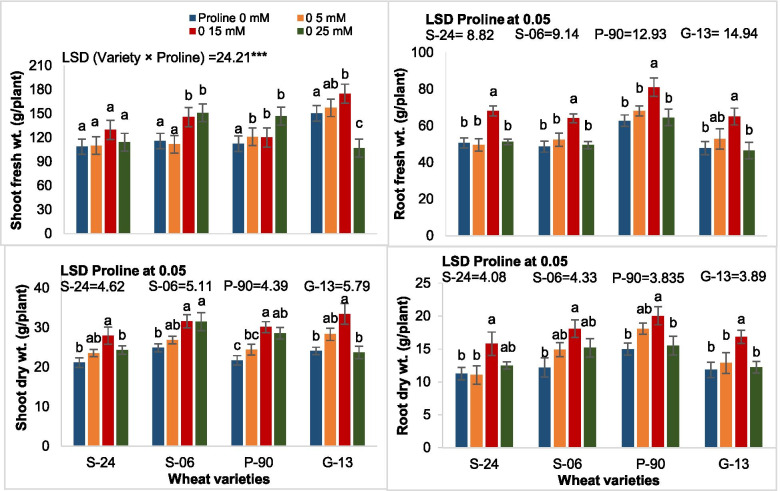
Fresh and dry weight of shoots and roots of plants of four wheat varieties raised from seeds primed with 0, 5, 15 and 25 mM proline (*n* = 3)

Seed priming with proline caused a significant effect on fresh and dry weights of shoots and roots of all four wheat varieties. Maximum increase in fresh and dry weights of shoots and roots was found in plants raised from seed priming with 15 mM proline. Varieties also differed significantly in all these growth attributes. Variety Galaxy-13 followed by Sehar-06 showed a maximal increase in shoot fresh weight at 15 mM proline. However, improving effect of 15 mM proline on shoot fresh weight was not observed in var. S-24. Since the interaction term var. x proline was only significant for shoot fresh weight, means of each variety at each proline level cannot be compared for other growth attributes. It is therefore, a separate one-way ANOVA for each variety was carried out followed by comparison of means with LSD for proline. Seed priming with 15 mM proline caused a maximum increase in shoot dry weight of all four wheat varieties. However, seed priming with 25 mM proline had a similar increasing effect on shoot dry weight of var. Sehar-06, whereas this dose of proline did not change the shoot dry weight of var. S-24 and Galaxy-13. Similarly, seed priming with 15 mM proline caused a maximal increase in root fresh and dry weight of all four wheat varieties. However, maximum increase in root fresh and dry weight was found in variety Pasban-90 (Fig. 1).

**Fig. 2:**
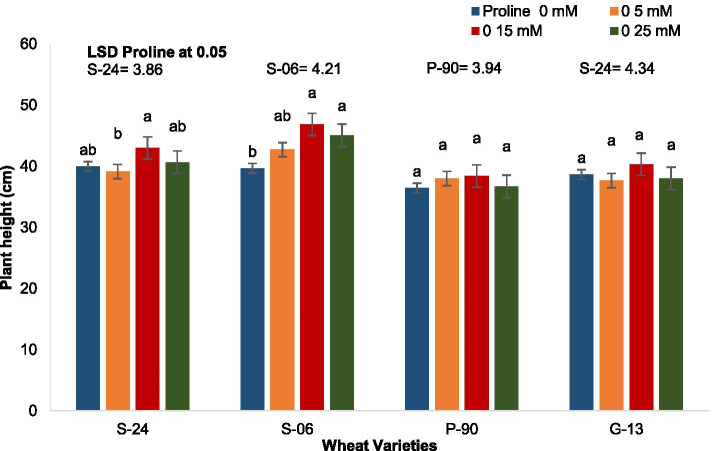
Height of plants of four wheat varieties raised from seeds primed with 0, 5, 15 and 25 mM proline (*n* = 3)

Analysis of variance of the data for plant height showed that seed priming with proline caused a significant effect on plant height and varieties differed in this attribute at different proline treatments. Seed priming with proline did not change the plant height of var. Pasban-90 and Galaxy-13, whereas it increased the plant height of var. S-24 and var. Sehar-06. Proline treatment had greater increasing effect on plant height (21%) of var. Sehar-06 than that in var. S-24. In addition, effect of 15 and 25 mM proline on plant height of Sehar-06 was similar (Fig. 2). Results for 2-way completely randomized ANOVA are presented in Table 1.

**Table 1:** Mean square values from ANOVA for shoot fresh weight, shoot dry weight, root fresh weight, root dry weight and plant height of four cultivars of wheat (*Triticum aestivum* L.) when raised from seed primed with proline (0, 5, 15 and 25 mM proline)

**SOV**	**df**	**Shoot fwt.**	**Shoot dwt**	**Root fwt.**	**Root dwt.**	**Plant height**
Proline	3	1003.8**	126.42***	783.43***	57.66***	26.81*
Variety	3	2132.0***	42.96**	705.24***	47.39***	87.55***
Proline ×Variety	9	1227.4***	12.78 ns	8.91 ns	2.27 ns	5.8 ns
Error	32	212.068	7.08	39.1	4.6	6.3

ns, non-significant; *, ** and *** significant at 0.05, 0.01 and 0.001 respectively

**Fig. 3:**
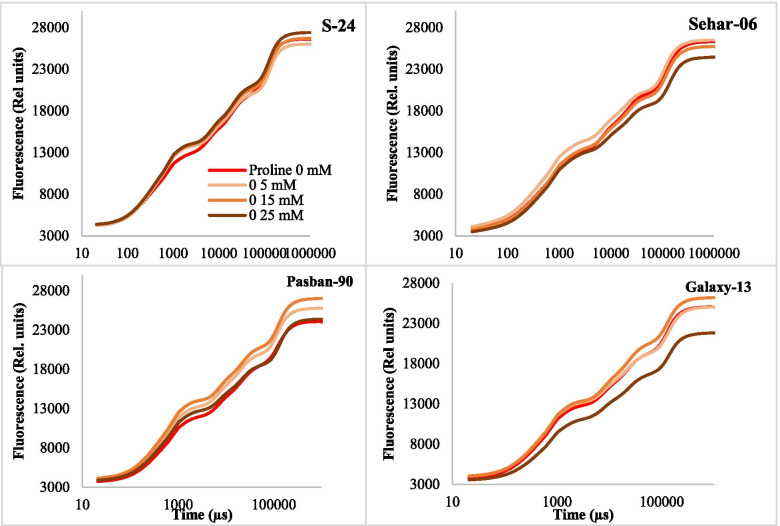
Chlorophyll fluorescence (Rel. units) of leaves of four wheat varieties (S-24, Sehar-06, Pasban-90 and Galaxy-13) raised from seeds primed with 0, 5, 15 and 25 mM proline

The data presented for raw OJIP curves of four wheat varieties as influenced by seed priming with different concentrations of proline (Fig. 3). Seed priming with proline did not change the Fo but changed fluorescence at J, I and P steps to varying extent in all four wheat varieties examined in this study. Moreover, the pattern of change in fluorescence at different step was different varieties. For example, seed priming with proline increased the fluorescence level at J, I and P steps in var. S-24 and Pasban-90. However, the extent of increase in fluorescence was higher in var. Pasban-90. In contrast, in var. sehar-06 and Galaxy-13, the effect of seed priming with 15 mM proline increased the fluorescence at I and/or P steps. In addition, seed priming with 25 mM proline reduced the fluorescence at J-I and I-P phase in var. Sehar, while in var. Galaxy-13 it reduced at O-J, J-I and I-P phase substantially (Fig. 3).

**Fig. 4:**
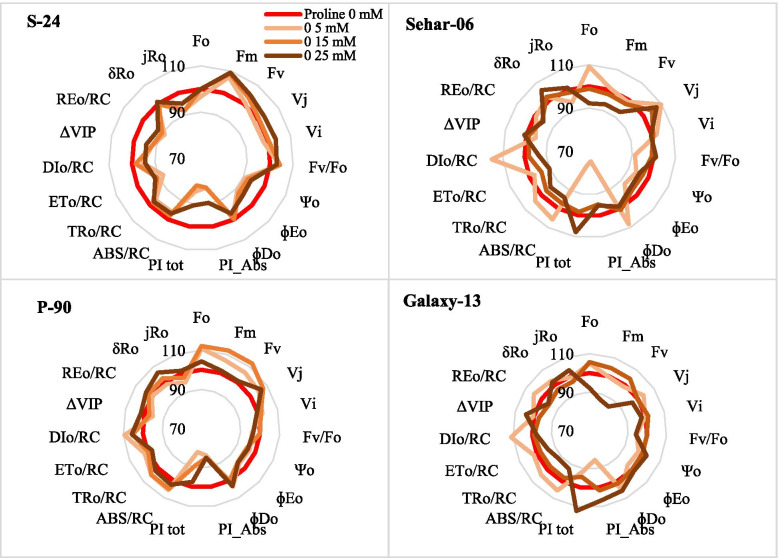
Different JIP-test parameters computed from OJIP raw curves data of plants of four wheat varieties (S-24, Sehar-06, Pasban-90, Galaxy-13) raised from seeds primed with 0, 5, 15 and 25 mM proline

The results for JIP-test parameters also showed that some of the parameters were substantially changed due to seed priming with proline, however, such changes were varietal specific. For example, seed priming with proline did not changed the basic fluorescence parameters (Fo, Fj, Fi, Fm, Fv) and ratios of fluorescence (Fv/Fm, Fm/Fo, Fv/Fo) in var. S-24, whereas in var. Sehar-06, only seed priming with 25 mM proline caused a significant decline in Fo. Likewise, seed priming with 25 mM proline caused a significant reduction in basic and ratio of fluorescence parameters. In contrast, in var. Pasban-90, seed priming with 5 and 15 mM proline enhanced Fo and Fj. Of relative variable fluorescence at J and I steps (V_J_ and V_I_), seed priming with proline significantly increased the V_J_ in var. S-24, Sehar-06 and Pasban-90, whereas V_J_ was reduced in var. Galaxy-13 at 25 mM proline (Fig. 3). Structural stability and functional activity of PSII is reflected by performance indices (PI_ABS_, PI_Tot_). Both performance indices were reduced due to seed priming with proline in all four wheat varieties, except that in Galaxy-13 where PI_ABS_ increased at 15 and 25 mM proline treatment (Fig. 4). Energy flux for absorbance (ABS/RC) and trapping (TRo/RC) remained unchanged due to seed priming with proline in plants of all four wheat varieties except those of Sehar-06 and Galaxy-13 plants raised from seeds primed with 25 mM proline, where both energy flux for absorbance and trapping decreased. Energy flux for electron transport (ETo/RC) decreased with seed priming with proline in var. S-24, Sehar-06 and Galaxy-13 while in var. Pasban-90 it remained unchanged. However, seed priming with proline reduced the energy flux for heat dissipation (DIo/RC) in var. S-24, whereas it increased in var. Pasban-90, Sehar-06 and Galxy-13 (Fig. 4).

To assess the changes electron transport flux from PSII to PSI, changes in ΔV_IP_, probability of electron transport flux from reduced QB to PSI end electron acceptors, and quantum efficiency of electron flux until PSI acceptors were calculated and found that all these JIP-test parameters were reduced due to seed priming with proline in only var. S-24. However, in other varieties either these remained unchanged or slightly increased.

**Fig. 5:**
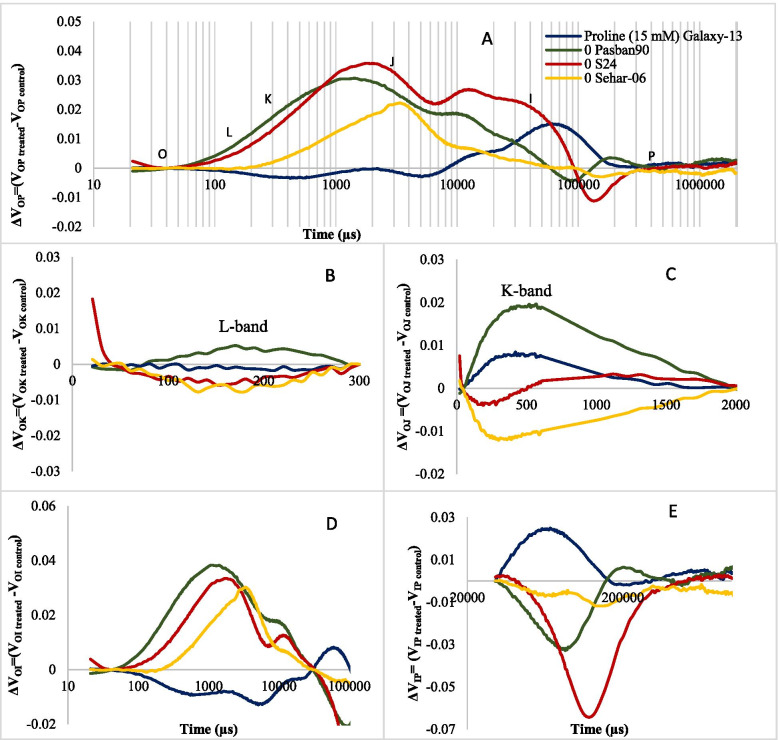
Differences in data curves normalized at O and P (**A**), O and K (**B**), O and J (**C**), O and I (**D**), I and P (**E**) points in four wheat varieties when primed with 15 mM proline

**Fig. 6:**
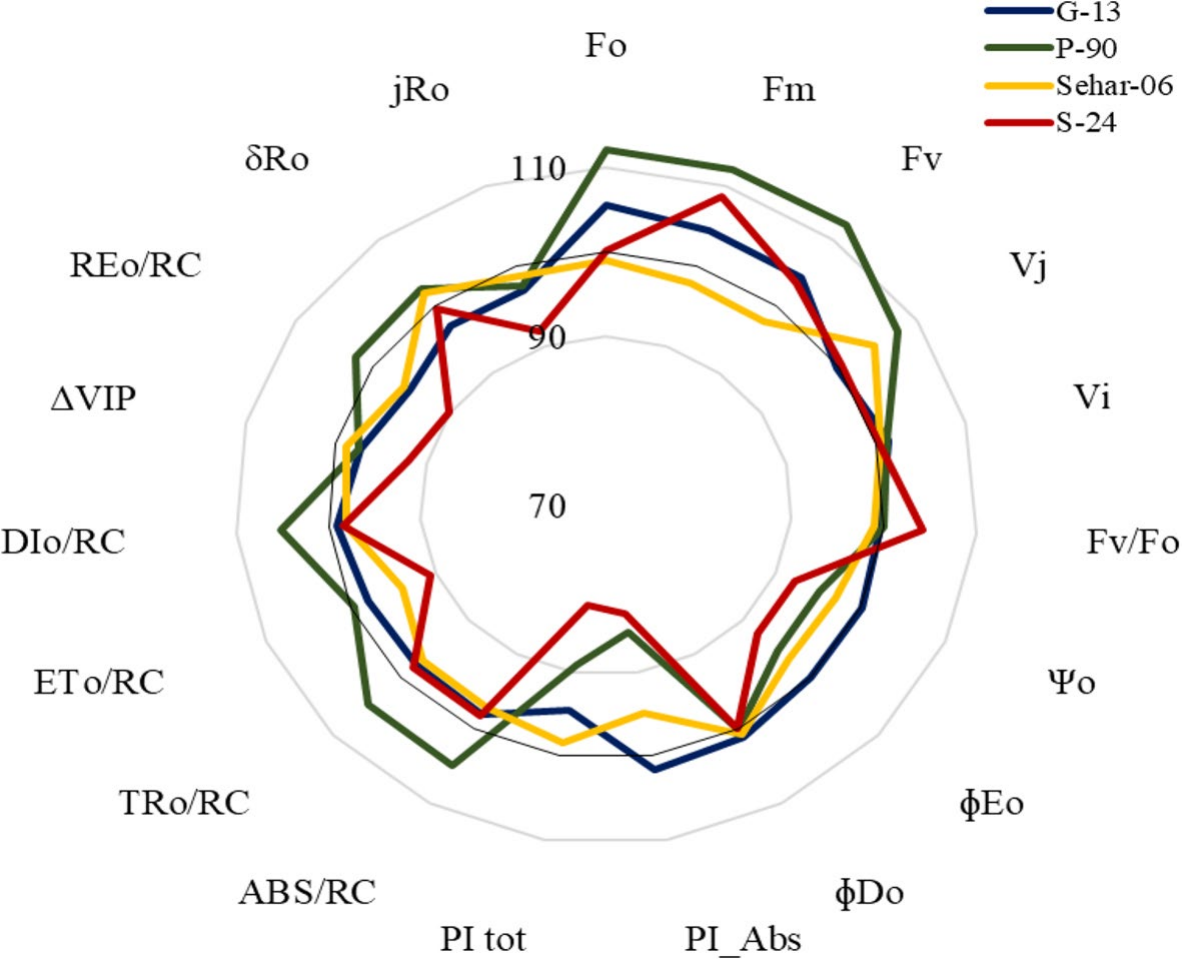
Comparison of JIP-test parameters of four wheat varieties (S-24, Sehar-06, Pasban-90, Galaxy-13) primed with 15 mM proline

Since the major changes occurred due to 15 mM proline seed priming treatment in all four wheat varieties, PSII activity in all four wheat varieties was compared. Differential kinetics of double normalized OJIP curves from O-P of four wheat varieties is presented in Fig. 6. Both varieties S-24 and Pasban-90 were significantly higher in differential double normalized fluorescence at L, K, J and I steps, whereas in var. Sehar-06 only J step appeared. In addition, positive band at P step appeared in var. Galaxy-13 (Fig. 5A). Differential kinetics for O-K as L band showed that there is no L-band appeared in all four wheat varieties (Fig. 5B). However, seed priming with 15 mM proline caused appearance of positive K band (O-J) in Pasban-90 and a negative K-band in Sehar-06, whereas there is no significant change in this attribute of plants of var. S-24 and Galaxy-13 (Fig. 5C). Differential kinetics at O-I phase indicated oxidation/reduction status of PQ pool and a positive peak was found in this region with seed priming with 15 mM proline in the three wheat varieties S-24, Sehar-06 and Pasban-90, whereas it almost unchanged in Galaxy-13 (Fig. 5D). The differential kinetics at I-P, which indicated electron transport flux from reduced PQ pool to PSI end electron acceptors, reduced due to seed priming with 15 mM proline in var. S-24, Sehar-06 and Pasban-90, whereas it increased in Galaxy-13 (Fig. 5E).

**Fig. 7:**
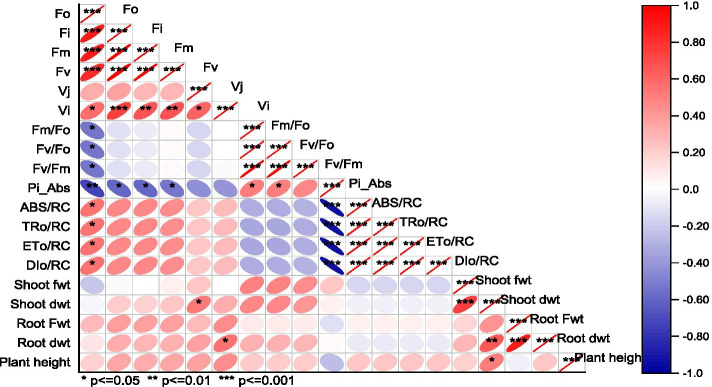
Correlation plot for assessment of the growth and stability of PSII in proline primed plants of different wheat varieties

Changes in JIP-test parameters of the four wheat varieties when primed with 15 mM proline was presented in Fig. 6. Among all basic fluorescence parameters, only Fo, Fm and Fv were significantly increased in var. Pasban-90 and Fm in var. S-24 (Fig. 6). Ratios of basic fluorescence parameters were almost remained unchanged in all four wheat varieties due to seed priming with 15 mM proline. Although quantum efficiencies of energy trapping and electron transport significantly decreased in var. Pasban-90 and S-24, energy flux for trapping remained unchanged due to proline seed priming in all four wheat varieties. However, energy flux for electron transport was significantly reduced in var. Sehar-06 and S-24. Likewise, seed priming with 15 mM proline reduced the PI_ABS_ in three wheat varieties i.e. S-24, Sehar-06, Pasban-90, whereas it remain unchanged in Galaxy-13. In addition, JIP-test parameters reflecting quantum efficiencies and probabilities with which PSII trapped excitons is transferred until PSI acceptors, and electron transport flux for PSI end electron acceptors per PSII all were significantly reduced on in var. S-24, whereas these remained almost unchanged in all other three wheat varieties (Fig. 6). Correlation matrix among different parameters is shown as Fig. 7.

## Discussion

Proline application through seed-priming, in this study, improved the plant growth of all four wheat cultivars. Proline-induced improvement in seedling growth of wheat cultivars can be explained in view of proline role in activation of seed metabolism for mobilization of food reserves toward growing plumule and radical during seed germination, which speed up seed germination and growth, particularly oxidative pentose phosphate pathway (OPPP) [18]. Our thorough studies suggested that the growth improvement due to seed priming with proline was concentration dependent and varietal specific, which is line with some of earlier studies with canola [2], wheat [19]. Seed priming with 15 mM proline was most effective in growth enhancement. Wheat variety Galaxy-13 followed by Sehar-06 were found to be most proline responsive. These results are in accordance with some other reports stating that growth improvement due to exogenous proline treatment was cultivar specific [2]. In addition, optimum proline dose for seed priming varied with type of species or even with type of cultivar of a same species. For example, 0.02% proline (~ 1.735 mM proline) as seed priming treatment was optimal for improving seed germination and seedling growth of rice, whereas higher concentration of proline (0.05% ~ 4.33 mM proline) proved to be inhibitory [20]. However, while working with canola cultivars Athar et al. [2] found that 5 mM proline was the most effective dose for growth improvement. In another study with wheat, it was found that seed priming with proline less than 10 mM did not improve the growth of wheat under non-saline or saline conditions [21], and they also reported that growth improvement in wheat occurred when seeds were primed with 10–30 mM proline. In comprehensive review literature, it has been reported that seed priming with 40 mM proline or greater than this caused adverse effects on plant growth in different crops [22–24]. These reports and results from the present study suggested that difference in optimum proline dose might have been due to differences in absorption of proline in seeds of different crops as well as genetic differences in seed metabolic machinery. This can be further explained by some of analogous studies in which it has been found that seed priming with salicylic acid improved the seed germination and seedling growth of *Solanum lycopersicum* (tomato), *Capsicum annuum* (Chili) but not in *Corylus avellana* [25, 26]. Studies revealed that applied salicylic acid bind with 7S globulin protein (vicilin proteins) having superoxide dismutase (SOD) activity and changes the redox state of the cell, which may act as down-stream signal for activation of various metabolic pathways for growth and stress tolerance [25, 26]. They also found that vicilin protein from *Corylus avellana* did not have functional salicylic binding pocket due to point mutation, and poor copper binding loop due to which it do not possess SOD activity. Thus, differential response of wheat varieties to seed priming with proline might have been due to proline induced differential activation of seed metabolism. Moreover, such proline induced growth response was dose specific, which can be explained as higher proline dose may initiate a signal of stress [27].

Previous studies suggested that exogenous application of proline as seed priming or as foliar spray improved the growth by improving photosynthetic activity [22, 28–31]. In the present study, priority targets of proline-induced PSII activity were assessed in four wheat varieties using fast chlorophyll *a* kinetic analysis i.e. OJIP analysis followed by JIP-test. From the ratios of basic chlorophyll fluorescence parameters such as Fv/Fo, it is clear that seed-priming with 15 mM proline improved the Fv/Fm and Fv/Fo in cvs. Sehar-06 and S-24. It may probably result from photoinhibition [32] occurring either due to decrease in its rate constant of photochemistry (leading to rise in Fo) or by increase in rate constant of non-radiative dissipation of excitation energy (leading to decrease in both Fo and Fm) [33]. These results and arguments are similar to those of [34] who also reported that application of higher proline dose caused a decline in Fv/Fm in maize plants.

Functional activity and structural stability of PSII is reflected by a multiple JIP-test parameter performance index (PI_ABS_). Performance index is product of reaction center density (light energy absorption), trapping and conversion efficiency of trapped excitons to electron transport. The improvement in PI_ABS_ in cv. Galaxy-13 due to 15 mM proline treatment and reduction in PI_ABS_ in other wheat cultivars might have been occurred due to reduction in any one or more than one component of PI_ABS_. These results are similar to the findings of previous studies with canola [35, 36] and wheat [37], who reported that PI_ABS_ is potential indicator of PSII activity for growth and stress tolerance, thus it can be used to screen genotypes with better photosynthetic capacity or growth under normal or stress conditions. Moreover, Mehta et al. [38] reported that the application of higher concentration of sucrose solution caused similar decrease in PI_ABS_ in wheat by decreasing efficiency of light reaction ((ɸP_o_)/(1-ɸP_o_)) and rate of biochemical reaction (ΨE_o_)/(1-ΨE_o_). In addition, such adverse effects were reversed by treating with water. These report and results from our study proposed that treatment with higher concentration of compatible solute might have adverse effects on energy fluxes in electron transport chain.

Seed priming with proline reduced energy flux for absorption per reaction centers, hence, showing that less energy was absorbed by antenna chlorophyll molecules in PSII [39] i.e. more active reaction centers were available as compared to the control. Moreover, energy flux for absorption and trapping become synchronized with energy flux for electron transport beyond Q_A_ by adjusting the energy flux for dissipation of heat as reported by Demitriou (2007). Thus, seed priming with 15 mM proline improved the primary photochemistry [40]. Furthermore, seed priming with higher concentration of proline increased the energy flux for absorption and quantum yield of primary photochemistry, but it decreased quantum yield of electron transfer which showed that treatment with higher dose caused inhibition of electron transfer beyond Q_A_ in these wheat varieties [41]. The increase in energy absorption and trapping with lower electron transport resulted in increase in energy dissipation in form of heat [40].

Raw OJIP curves and semi-quantitative analysis of normalized OJIP curves indicated that proline treatment changed fluorescence at J, I and P steps in all four wheat varieties but it did not cause a reduction Fv/Fm or photochemistry, which indicated that proline treatment caused substantial changes in energy fluxes at various points of PSII and/or electron transport chain. Increase in fluorescence at O-K phase or L-band in cvs. Pasban-90 and S-24 (Fig. 5A and B) indicated that proline treatment caused (to some extent) loss of energetic connectivity of light harvesting complex (LHCII) and reaction center in these two wheat varieties or in other words absorbed photons by antennae were poorly managed by the PSII reaction centers [41–43]. In addition, proline treated plants of var. Galaxy-13 were better in energetic connectivity between LHCII and PSII reaction center than in other wheat varieties.

Proline treatment caused an increase fluorescence at J step and appearance of positive K-band in the three wheat varieties S-24, Sehar-06 and Pasban-90 (except in Galaxy-13) indicating proline treatment might have caused a disturbance to some extent at donor or acceptor end of PSII. This can be explained as imbalance in electron flow from oxygen evolving complex (OEC) to PSII reaction center, acceptor end of PSII and its subsequent transfer towards PSI as discussed elsewhere [41, 42]. However, proline induced changes in fluorescence at J-I phase particularly in Pasban-90 and S-24 depicted increased re-oxidation of Q_A_ by Q_B_ and other electron acceptors [40, 44]. Negative bands for changes in O-I region of proline treated wheat plants in three wheat varieties except than in Galaxy-13 also indicated that incidents starting from exciton trapping to PQ reduction were faster in proline treated plants than in control, particularly in Galaxy-13. Similar results has already been observed in canola plants treated with glycine betaine [42]. Interestingly, changes in fluorescence at I-P phase in proline treated plants of cv. S-24 showed a significant decline in capacity of PSI functionality (i.e., production of NADPH and CO_2_ fixation) [45]. Such decline in PSI functionality can be related to poor electron transport from PSII as reflected from reduced values of quantum efficiencies and probability with which trapped exciton by PSII is transferred to PSI end acceptors [46, 47].

## Conclusion

Seed priming with 15 mM proline proved to be optimum dose for growth improvement of all four wheat cultivars examined in this study. Higher dose of proline was not effective in improving growth of wheat plants. Maximum growth improvement due to seed priming with proline was found in var. Galaxy-13 followed by Sehar-06. Proline-induced growth improvement of wheat was positively associated with proline induced increase in primary photochemistry of PSII. Changes in primary photochemistry of PSII was due to better management of absorbed energy in electron transport by electron acceptors of electron transport, particularly those present at PSI end.

## Materials and methods

The experiment was conducted in natural conditions in the wire-net house of the Botanic Gardens of Bahauddin Zakariya University, Multan, Pakistan. The plastic pots (1.8 ft. in height and 1.2 ft. diameter) were filled with ordinary river sand washed with water. Seeds of four different wheat varieties; S-24, Sehar-06, Galaxy-13 and Pasban-90, were obtained from Ayyub Agriculture Research Center Faisalabad, Pakistan. The S-24 is claimed to be high yielding-salt tolerant, and other three wheat varieties are high yielding and cultivated on large areas of Pakistan in the recent past. The seeds of all varieties were primed with four different concentrations of proline (Merck) i.e. 0, 5, 15 and 25 mM for 12 h. Seeds were slightly dried and sown in pots filled with sand at equal distances. Seedlings were nurtured with Hoagland nutrient solution every week. After 1 week, seedlings were thinned to six per pot. Plants were allowed to grow in full daylight for 4 weeks. Fast chlorophyll a kinetic analysis or OJIP curves were recorded on dark adapted leaves following Strasser [48]. The detailed protocol is given below in a separate section. Plants were harvested at 6-week-old stage. Before harvest, plant height of each variety was recorded. At the time of harvest, plants were carefully uprooted from pots and roots were washed. Plants of each wheat variety were separated into shoots and roots and their fresh weights were recorded using sensitive balance. Samples were then oven dried at 70 C for 3 days and their dry weights were recorded.

### Chlorophyll *a* fluorescence

Chlorophyll *a* fluorescence (OJIP curves) analysis was performed using handheld continuous chlorophyll fluorescence meter or non-modulated fluorometer (PAR-Fluorpen FP 100-Max-LM). Leaves were dark adapted for 30 min. A weak light (1 μmol m^− 2^ s^− 1^) was applied to measure Fo and then a saturation pulse of 3000 μmol m^− 2^ s^− 1^ was applied to measure fluorescence over 1 s. Fluorescence induction curve (OJIP) was plotted on log scale. OJIP curve, known as Katusky curves, divides whole processes occurring at PSII complexes in four steps: O, J, I and P. From OJIP curves, semi-quantitative analysis was also carried out following Kalaji et al. 2016. The raw OJIP-curves were normalized and double normalized for fine depiction of variations among different treatments. Formulas used were: Fo_Norm_ = (Ft/Fo), Fm_Norm_ = (Ft/Fm), V_OP_ = (Ft-Fo/Fm-Fo), V_OK_ = (Ft-Fo/Ft_300_-Fo), V_OJ_ = (Ft-Fo/Fj-Fo), V_OI_ = (Ft-Fo/Fi-Fo), and V_IP_ = (Ft-Fi/Fp-Fi). Differences in these curves (treated-control) were plotted to analyze differential response of varieties to variations in proline dose. Initial shape of curve depends upon O-J (L-band; PSII grouping) and K-band (balance between electron donation from OEC and electron acceptance from Q_A_-). **J-I** phase of the curve shows reduction of secondary electron acceptor Q_B_, plastoquinone, cytochrome b6f and plastocyanin. **I-P** phase shows reduction of electron transporters of PSI acceptor side. Maximum fluorescence intensity or point P shows saturation of all reaction centers when strong light ensures a balance of oxidation and reduction [43]. This phase also provides an insight into cyclic electron flow and ultimately ratio of ATP and NADPH [49]. **O-P** is also known as relative variable fluorescence i.e. V=Variable fluorescence/Maximal variable fluorescence [47].

JIP-test parameters were calculated following Strasser [50]. JIP-test is based upon basic theory of energy flow across thylakoid membranes and total energy inflows and outflows from light harvesting complex. The probable distribution of absorbed energy between PSII complexes helps to study any changes in structure of PSII. Basic fluorescence parameters and ratio of basic fluorescence parameters were also recorded as:

**Table:** 

**Symbol**	**Formula**	**Description**
**Fo**	Fluorescence at 0.05 ms	Fluorescence at initial point
**Fk**	Fluorescence at 0.3 ms	Fluorescence at K point
**Fj**	Fluorescence at 2 ms	Fluorescence at J point
**Fi**	Fluorescence at 30 ms	Fluorescence at I point
**Fm**	Fluorescence at 300 ms	Fluorescence at P point
**Fv**	Fv = Fm-Fo	indicates the variation in fluorescence from initial to final point of OJIP transient curve
**PI**_**total**_	=PI_ABS_ × δRo / (1- δRo)	
**Fv/Fm**		quantum yield of primary PSII photochemistry at t = 0
**ɸEo**	=ETo/ABS or ϕETo = 1-Fj/Fm = ϕPo∙(1-Vj)	the quantum yield of electron transport
**Vj**	=(Fj-Fo)/(Fm-Fo)	relative variable fluorescence at t = 2 ms
**Vi**	=(Fi-Fo)/(Fm-Fo)	relative variable fluorescence at t = 30 ms.
**ψEo**	=(1-Vj)	efficiency/probability with which an electron trapped in PSII RC is transferred beyond Q_A_.
**RC/ABS**	=ϕPo∙Vj/Mo	number of Q_A_ reducing RCs per PSII antenna chlorophyll.
**TRo/RC**	=Mo∙(1/Vj)	trapped energy flux per RC at t = 0
**ETo/RC**	=Mo∙(1/Vj) ∙ ψEo)	electron transport flux further than Q_A_- per RC
**PI**_**ABS**_	=(RC/ABS)∙(ϕPo/1-ϕPo)∙(ψEo/1- ψEo)	performance index (potential) for energy conservation from photons absorbed by PSII antenna to the reduction of Q_B_
**ΔVIP**	= 1- Vi	Changes in IP phase
**REo/RC**	=Mo(1/Vj)(1-Vi)	energy flux reducing end electron acceptor of PSI
**dRo**	=REo/ETo = (1-Vi)/(1-Vj)	probability to reduce E-acceptors at acceptor side of PSI
**jRo**	=[1- (Fo/Fm)] × (1-Vi)	quantum yield to reduce E-acceptors at acceptor side of PSI

Strasser et al. [50]. It also relates reduction of intersystem electron acceptors and is based upon density of active centers trapping probability and efficient electron transmittance beyond Q_A_ [50, 51].

### Statistical analysis

The data obtained were subjected to two-way analysis of variance. For ANOVA, CoStat 6.5 was used (CoHort, California, USA). If the interaction term was significant, means were compared with LSD. The JIP-test parameters were transformed a percent of control and plotted as radar plot. Originpro-20 was used for deriving Pearson correlation coefficients to find out correlation among various parameters of growth and OJIP.

## Data Availability

The data and materials are included this article in form of graphs. He data is purely original and taken from experiment carried out by the authors.

## Abbreviations

S-06: Sehar-06
P-90: Pasban-90
G-13: Galaxy-13
P-5: Proline 5 mM
P-15: Proline 15 mM
P-25: Proline 25 mM
Fv/Fm: Quantum yield
PI_ABS_: Performance index
